# Removal of the Clavicle in a State of Osteo-Sarcoma

**Published:** 1833

**Authors:** 


### II. ANALYTICAL.

ART. I. *Removal of the Clavicle in a state of Osteo-Sarcoma.*—The distinguished professor of Anatomy and Surgery in Harvard University, Dr. Warren, has lately performed this operation. The patient, a man twenty-four years old, apparently in consequence of lifting a heavy weight, experienced, after several months, an osteo-sarcoma of the right clavicle.

The following is the professor's account of the operation, as published in the American Journal for November:

"The patient being placed on a table, the shoulders elevated, an incision was made from the acromial extremity of the clavicle to the sternal extremity of the clavicle of the opposite side. This was crossed by an incision at right angles with it, beginning just below the middle of the sterno-mastoid muscle, and extending to the face of the pectoralis muscle below the middle of the clavicle. The four flaps were then dissected from the surface of the tumour. Next the outer extremity of the clavicle was laid bare, by dissecting the deltoid muscle from its anterior edge, and the trapezius from its posterior edge, and the division of the coraco-clavicular ligaments. An eyed probe, armed with a ligature, was then passed under the clavicle, and the ligature being attached to a chain saw, this was drawn under, and the clavicle sawed through.

The separation of the tumour now began.

A strong ligature being passed around the outer extremity of the divided clavicle, the tumour was partially moved by it, so as to give tension to the surrounding soft parts. The pectoralis major muscle was cut through, and dissected from the lower edge of the tumour, and drawn so as to expose the pectoralis minor and the cephalic vein. Now the dissection extending under the tumour, the subclavian muscle was distinguished and dissected from the tumour at its outer part, but at the sternal part it was lost in the tumour, where of course the dissection proceeded over the surface of the subclavian vein. An adhesion of the tumour to the second rib, in which it was imbedded, prevented a perfect separation at this part till the close of the operation.

The next step was to divide the attachments to the upper or cervical edge of the tumour. First the posterior external jugular vein was divided and tied: it was filled with a dense lymph, and discharged no blood. Next the sterno-mastoid muscle was cut across, and the sheath of the cerebral blood-vessels exposed. The internal jugular vein was perceived to pass from the neck into the substance of the tumour in such way as to render it difficult to dissect without dividing it. This was however accomplished, and then the carotid artery and par vagum nerve presented themselves. On reaching the internal extremity of the tumour, the anterior external jugular vein was found imbedded in it. This was filled with solid lymph, and was tied, as it would not have been safe to have left it without a ligature.

Nothing now remained to be done, but to separate the sternal end of the tumour from the corresponding part of the jugular and subclavian veins. By great caution this was safely accomplished, and the tumour removed. When this was effected, the whole extent of the subclavian vein was exposed—the lower part of the jugular vein and the par vagum nerve. These being put in motion by the pulsation of the subclavian and the carotid arteries, and the arteria innominata, presented a formidable appearance.

Little blood was lost in the operation. Only one or two arteries were tied, and the veins specified above. The flaps were brought over and retained by three sutures and adhesive plasters, so as to cover the wound perfectly.

The patient's appearance after the operation was good, and by a recurrence to the Surgical Diary, it appears his symptoms were most favorable to the thirteenth day. He was then able to sit up; had appetite; took small quantities of solid food, and had every promise of a favorable termination of his case.

The wound united to a considerable extent by the first intention, and the united part went on favorably. On the thirteenth day he was affected with chills, pain in the epigastric region, and his pulse rose from 80 to 112. These symptoms were followed by a nervous agitated state, and eventually by a slight delirium, with no other local phenomena than these above-mentioned.

On some days he appeared better, and in a fair way to recover, and this hope was not abandoned till the day before his death, which occurred on the 8th day of December, in the fourth week from the operation."

The patient was of an irritable and scrophulous habit; and to this the professor ascribes the fatal termination of the case.

